# Effect of population-based antenatal screening and treatment of genitourinary tract infections on birth outcomes in Sylhet, Bangladesh (MIST): a cluster-randomised clinical trial

**DOI:** 10.1016/S2214-109X(18)30441-8

**Published:** 2018-12-13

**Authors:** Anne CC Lee, Luke C Mullany, Mohammad Quaiyum, Dipak K Mitra, Alain Labrique, Parul Christian, Parvez Ahmed, Jamal Uddin, Iftekhar Rafiqullah, Sushil DasGupta, Mahmoodur Rahman, Emilia H Koumans, Salahuddin Ahmed, Samir K Saha, Abdullah H Baqui

**Affiliations:** a Department of Pediatric Newborn Medicine, Brigham and Women's Hospital, Boston, MA, USA; b International Center for Maternal and Newborn Health, Johns Hopkins Bloomberg School of Public Health, Baltimore, MD, USA; c Department of International Health, Johns Hopkins Bloomberg School of Public Health, Baltimore, MD, USA; d International Center for Diarrheal Diseases—Bangladesh, Center for Reproductive Health, Dhaka, Bangladesh; e North South University, Dhaka, Bangladesh; f Independent University, Bangladesh, Dhaka, Bangladesh; g Bill & Melinda Gates Foundation, Seattle, WA, USA; h Department of Microbiology and Immunology, University of Mississippi Medical Center, Jackson, MS, USA; i Centers for Disease Control and Prevention, Atlanta, GA, USA; j Johns Hopkins University—Bangladesh, Dhaka, Bangladesh; k Child Health Research Foundation, Department of Microbiology, Dhaka Shishu Hospital, Dhaka, Bangladesh

## Abstract

**Background:**

One-third of preterm births are attributed to pregnancy infections. We implemented a community-based intervention to screen and treat maternal genitourinary tract infections, with the aim of reducing the incidence of preterm birth.

**Methods:**

We did an unblinded cluster-randomised controlled trial in two subdistricts of Sylhet, Bangladesh. Clusters were defined as the contiguous area served by a single community health worker, and each cluster comprised several contiguous villages, contained roughly 4000 people, and had about 120 births per year. Eligible participants within clusters were all ever-married women and girls of reproductive age (ie, aged 15–49 years) who became pregnant during the study period. Clusters were randomly assigned (1:1) to the intervention or control groups via a restricted randomisation procedure. In both groups, community health workers made home visits to identify pregnant women and girls and provide antenatal and postnatal care. Between 13 and 19 weeks' gestation, participants in the intervention group received home-based screening for abnormal vaginal flora and urinary tract infections. A random 10% of the control group also received the intervention to examine the similarity of infection prevalence between groups. If present, abnormal vaginal flora (ie, Nugent score ≥4 was treated with oral clindamycin (300 mg twice daily for 5 days) and urinary tract infections with cefixime (400 mg once daily for 3 days) or oral nitrofurantoin (100 mg twice daily for 7 days). Both infections were retreated if persistent. The primary outcome was the incidence of preterm livebirths before 37 weeks' gestation among all livebirths. This trial is registered with ClinicalTrials.gov, number NCT01572532. The trial is closed to new participants, with follow-up completed.

**Findings:**

Between Jan 2, 2012, and July 28, 2015, 9712 pregnancies were enrolled (4840 in the intervention group, 4391 in the control group, and 481 in the control subsample). 3818 livebirths in the intervention group and 3557 livebirths in the control group were included in the primary analysis. In the intervention group, the prevalence of abnormal vaginal flora was 16·3% (95% CI 15·1–17·6) and that of urinary tract infection was 8·6% (7·7–9·5). The effective coverage of successful treatment in the intervention group was 58% in participants with abnormal vaginal flora (ie, abnormal vaginal flora resolved in 361 [58%] of the 622 participants who initially tested positive), and 71% in those with urinary tract infections (ie, resolution in 224 [71%] of the 317 participants who initially tested positive). Overall, the incidence of preterm livebirths before 37 weeks' gestation did not differ significantly between the intervention and control groups (21·8% *vs* 20·6%; relative risk 1·07 [95% CI 0·91–1·24]).

**Interpretation:**

A population-based antenatal screening and treatment programme for genitourinary tract infections did not reduce the incidence of preterm birth in Bangladesh.

**Funding:**

Eunice Kennedy Shriver National Institute of Child Health and Human Development and Saving Lives at Birth Grand Challenges.

## Introduction

Globally, an estimated 14·8 million infants were born preterm (<37 weeks' gestation) in 2014, and preterm birth rates are increasing in many countries.^1^ More than 90% of preterm births occur in low-income and middle-income countries (LMICs),^2^ where access to, and quality of, antenatal, intrapartum, and postnatal care vary. Complications from preterm birth are now the leading cause of child mortality and account for 1 million neonatal deaths annually.^3^ Survivors of preterm birth have increased risk of neurodevelopmental impairment, stunting, and chronic disease.^4^, ^5^ Thus, effective strategies are needed for primary prevention of preterm birth in LMICs.

**Research in context**

**Evidence before this study**

A third of preterm births are attributed to pregnancy infections, which are commonly undetected and untreated in low-income and middle-income countries. Abnormal vaginal flora (bacterial vaginosis and intermediate flora—ie, Nugent scores ≥4) is significantly associated with preterm birth. However, routine antenatal screening is not recommended in the general obstetric population. In a Cochrane review, screening and treatment of asymptomatic bacterial vaginosis in the general obstetric population did not reduce the risk of preterm birth (pooled risk ratio 0·88 [95% CI 0·71–1·09]; n=6491 from 13 trials). However, in pooled analysis of two trials (n=894 women) in the UK that targeted all abnormal vaginal flora, treatment with clindamycin significantly reduced preterm births before 37 weeks (risk ratio 0·53 [95% CI 0·34–0·84]) compared with treatment with placebo. Antenatal screening and treatment of bacteriuria in pregnancy is recommended by WHO. There is strong evidence that such screening and treatment reduces maternal pyelonephritis. However, with respect to birth outcomes, Cochrane grades the quality of evidence as low for preterm birth (risk ratio 0·27 [95% CI 0·11–0·62]; n=242 participants) and low birthweight (risk ratio 0·64 [0·45–0·93]; n=1437 participants). Most studies were done in the 1960s or 1970s, and this strategy of antenatal screening and treatment of bacteriuria has not been rigorously assessed in a low-middle-income country.

**Added value of this study**

We studied the effect of a population-based antenatal screening and treatment programme for abnormal vaginal flora and urinary tract infections on the population incidence of preterm livebirth in Sylhet, Bangladesh. Effective coverage of successful treatment was low (58% for abnormal vaginal flora and 71% for urinary tract infections), despite repeated treatment of persistent infections. The incidence of preterm livebirths before 37 weeks' gestation did not differ significantly between the intervention (21·8%) and control (20·6%) groups (risk ratio 1·07 [95% CI 0·91–1·24]). In post-hoc analyses, participants with persistent abnormal vaginal flora were significantly more likely to deliver preterm than those without abnormal vaginal flora, whereas the risk among those with abnormal vaginal flora who were cured with antibiotics was similar to that in uninfected peers.

**Implications of all the available evidence**

In rural Bangladesh, a population-based antenatal screening and treatment programme for abnormal vaginal flora and urinary tract infections in early pregnancy did not reduce the incidence of preterm birth. Effective coverage rates of successful treatment for both infections were low. Our findings highlight the importance of increasing antibiotic resistance, and the need to better describe the microbiological nature of abnormal vaginal flora and to identify treatments with better clinical efficacy for both infections. Additional research is needed to study the role of other infections and risk factors for preterm birth in low-income and middle-income countries. Comprehensive and innovative approaches are needed to address maternal infections in pregnancy in low-income and middle-income countries to prevent preterm birth and help to reduce the large burden of preterm-birth-related morbidity and mortality.

Strong evidence supports the role of maternal infections in preterm birth, with 30–50% of preterm births attributed to maternal infections in pregnancy.^6^, ^7^, ^8^, ^9^ Ascending infection from the lower genital tract leads to amniotic fluid infection and inflammation, and could result in uterine contractions, cervical ripening, and premature rupture of membranes.^9^, ^10^ In LMICs, maternal genitourinary tract infections are prevalent, inadequately diagnosed and treated, and often asymptomatic. Such infections during pregnancy have been significantly associated with miscarriage, stillbirth, preterm birth, fetal growth restriction, neonatal and puerperal sepsis, and neonatal encephalopathy.^11^, ^12^, ^13^, ^14^

Bacterial vaginosis, the most prevalent reproductive tract infection in pregnancy globally, is consistently associated with preterm birth.^15^, ^16^, ^17^ It is a polymicrobial syndrome triggered by imbalance in concentrations of endogenous vaginal microflora and overgrowth of anaerobic species.^18^ The term abnormal vaginal flora includes bacterial vaginosis and an earlier transitional state towards bacterial vaginosis, which is known as intermediate flora.^19^ Screening and treatment of bacterial vaginosis has no effect on preterm delivery in low-risk pregnancies,^20^ and the US Preventive Services Task Force advises against routine screening in asymptomatic pregnant women.^21^ However, two randomised placebo-controlled trials^22^, ^23^ in the UK in women with abnormal vaginal flora showed that treatment in early pregnancy (ie, <24 weeks), before the sealing of the amniotic membranes, reduced the incidence of preterm birth. These trials separately examined oral^22^ and intravaginal^23^ treatment with clindamycin, demonstrating substantive (>50%) reductions in risk of preterm birth.

Urinary tract infections affect an estimated one in four pregnant women in LMICs.^24^, ^25^ In the USA in the 1960s, before routine screening was introduced, 40% of pregnant women with untreated bacteriuria developed pyelonephritis,^26^ and 30–50% of women with pyelonephritis delivered preterm.^27^, ^28^, ^29^ In a meta-analysis,^30^ pregnant women with asymptomatic bacteriuria had a two-times higher risk of preterm delivery (relative risk 2·00 [95% CI 1·43–2·77]) compared with those without bacteriuria. Although evidence for the effect of treatment of asymptomatic bacteriuria on preterm birth risk is weak,^31^ treatment significantly reduces maternal pyelonephritis^32^ and risk of low birthweight, and screening and treatment are recommended in pregnancy by the Infectious Diseases Society of America,^33^ Canadian Task Force on Preventive Care,^34^ and the UK National Institute for Health and Care Excellence.^35^ In 2016, WHO made context-specific antenatal care recommendations for screening and treatment of asymptomatic bacteriuria in LMICs.^36^

We hypothesised that abnormal vaginal flora and urinary tract infections were prevalent and not adequately addressed in the rural district of Sylhet in Bangladesh. In view of strong observational data, promising preliminary intervention studies of treatment of abnormal vaginal flora,^22^, ^23^ and an absence of effective primary prevention measures in low-income and middle-income settings, we aimed to assess the effect of a community-based antenatal screening and treatment programme for abnormal vaginal flora and urinary tract infections in early pregnancy (13–19 weeks) on population-level rates of preterm birth in Sylhet.

## Methods

### Study design, setting, and participants

The Maternal Infection Screening and Treatment (MIST) study was an unblinded cluster-randomised controlled trial done in two subdistricts (Zakiganj and Khanaighat) of Sylhet district, Bangladesh. A detailed trial protocol was previously published.^37^ Additional methods are included in the appendix.

The trial was done at the Projahnmo research site—a research partnership between Johns Hopkins University (Baltimore, MD, USA), the Bangladesh Ministry of Health and Family Welfare (Dhaka, Bangladesh), Shimantik (a non-governmental organisation; Kaliganj, Sylhet, Bangladesh), the Child Health Research Foundation (Dhaka, Bangladesh), and Brigham and Women's Hospital (Boston, MA, USA)—which was established in 2001 (appendix). The study area has been previously described,^38^ and was chosen because access to health care there is poor but need is high. It has one of the highest rates of neonatal mortality in Bangladesh. The study area consisted of 24 clusters, which were defined as the contiguous area served by a single community health worker and four or five village health workers (each cluster comprised several contiguous villages, contained roughly 4000 people, and had about 120 births per year per cluster). Coverage of antenatal and intrapartum care within the formal government health system was low in the population during the study period, with only around half of mothers receiving any antenatal care within the health system, and around 80% delivering at home. Screening and treatment for genitourinary tract infections were not standard of antenatal care in this region.

Before the study, all households were mapped by geographical information systems, and all ever-married women and girls of reproductive age (ie, aged 15–49 years) were enumerated. This list was updated bimonthly through the duration of the study. All married women and girls of reproductive age in the study areas were provided with home calendars and instructed to circle the first day of each menstrual cycle. Health workers completed monthly household pregnancy surveillance visits, and a urine pregnancy test was done in the home if the last menstrual period was more than 4 weeks ago. All women and girls in the study area whose pregnancy was detected before 19 weeks' gestation were eligible to enrol in the study. Women were excluded from the study if they were unsure of the date of their last menstrual period (because of lactational amenorrhea, recent discontinuation of contraception, or irregular menses) or had severe chronic disease.

The study protocol was approved by the institutional review boards of Johns Hopkins Bloomberg School of Public Health (Baltimore, MD, USA), the International Centre for Diarrhoeal Disease Research, Bangladesh (Dhaka, Bangladesh), and Partners HealthCare (Boston, MA, USA). A data and safety monitoring board reviewed the study procedures at the start of the trial and interim analysis. Participants provided oral consent to community health workers.

### Randomisation and masking

Projahnmo project health workers enrolled participants. Eligible clusters were randomly assigned (1:1) to either screening and treatment of genitourinary tract infections (intervention group) or standard care (control group) via a restricted randomisation procedure at Johns Hopkins University operated by LCM, the study statistician. All possible randomisation sequences were generated, allocating 12 clusters to the intervention group, and 12 clusters to the control groups. On the basis of previous data,^39^ we restricted the eligible sequences to those in which intervention:control ratios for predicted preterm birth incidence were within 0·975–1·025. Subsequently one sequence was randomly selected. To compare the baseline prevalence of infections between intervention and control clusters, 10% of participants in the control clusters were randomly selected to receive the intervention.^37^ Because of the nature of the intervention, the trial intervention could not be masked to either investigators (including the study statistician) or participants. However, health workers and study participants were not aware of study hypotheses and outcome measures.

### Procedures

Community health workers provided basic home-based antenatal care (between 13 and 19 weeks' and 28 and 32 weeks' gestation)^37^ in all study areas. Participants in the intervention clusters and the control subsample also underwent initial screening for genitourinary tract infection and subsequent treatment, if needed, was provided during home visits between 13 and 19 weeks' gestation (appendix).^37^ A single self-administered vaginal swab was collected by participants at these home visits. Community health workers then rolled the swab onto a glass slide, that was later gram stained and Nugent scored in the Sylhet laboratory (appendix).^40^ Similar methods have been used with high acceptability and specimen quality in diverse patient populations, including in Bangladesh.^41^, ^42^, ^43^ A clean-catch midstream urine specimen was collected at this visit, and urine culture was done (the process was repeated if cultures were contaminated). For laboratory quality control, a random 5% of vaginal specimens were independently scored by an external expert (AL), and 5% of urine culture isolates were confirmed at a reference laboratory (Dhaka Shishu Hospital, Dhaka, Bangladesh).

Women and girls with abnormal vaginal flora or urinary tract infections were provided with antibiotic treatment on the basis of laboratory results, irrespective of whether or not they were symptomatic. Those with clinical symptoms were further referred to the subdistrict hospital for clinical assessment by a health-care provider.^37^ Abnormal vaginal flora was treated with 300 mg clindamycin orally twice daily for 5 days.^22^ Urinary tract infections were initially treated with 400 mg cefixime once daily for 3 days, but treatment was changed in October, 2012, to 100 mg nitrofurantoin orally twice daily for 7 days after a high prevalence of cefixime resistance was detected. The first dose was directly observed, and compliance with the full course was assessed by pill count by community health workers. The first test-of-cure specimen was obtained 1 week after treatment for urinary tract infections and 3 weeks after treatment for abnormal vaginal flora.^22^ Participants with persistent infection were retreated (abnormal vaginal flora with clindamycin as per Lamont and colleagues' methods,^22^, ^23^ urinary tract infections on the basis of antibiotic sensitivity). A final test-of-cure specimen was obtained, and those with persistent urinary tract infections were referred to Sylhet Medical College Hospital for assessment and management.

Community health workers were notified of all pregnancy outcomes by village health workers and completed home visits as soon as possible after pregnancy outcome. They gathered data for birth outcomes, antepartum and intrapartum complications, and maternal or neonatal morbidity, and also did a neonatal assessment. Community health workers completed postnatal home visits on the first, third, seventh, and 28th day after birth. Follow-up continued until Feb 29, 2016.

### Outcomes

The primary outcome was preterm livebirths before 37 weeks' gestation. Gestational age was based on the first day of the last menstrual period.^44^ Secondary outcomes included all pregnancy outcomes before 37 weeks' gestation (including late miscarriage and preterm stillbirth), low birthweight (ie, <2500 g; weight measured within 72 h of birth), small for gestational age (weighing less than the 10% birthweight cutoff for gestational age and sex on the basis of the Intergrowth-21st standard^45^), maternal clinical urinary tract infection after 20 weeks' gestation, and neonatal mortality. For denominators for all births, we restricted pregnancy outcomes a priori to those of greater than 20 weeks' gestation, because our intervention was delivered up to 20 weeks' gestation. Full definitions of all outcomes, and a full list of secondary outcomes are in the appendix.

### Statistical analysis

We assumed that roughly 15% of women in this setting would have at least one genitourinary tract infection (either abnormal vaginal flora or urinary tract infections). Drawing upon available studies,^20^, ^21^ we assumed that the relative risk (RR) of preterm birth comparing infected to non-infected women was 2·5,^22^, ^23^ and that the true intervention effect would be a reduction in preterm birth of roughly 60% among infected women on the basis of effect sizes reported in two previous studies^22^, ^23^ that included women with abnormal vaginal flora. These assumptions would imply a population-level reduction in the proportion of preterm births of 18·4%, a level consistent with our a-priori established minimal range of public health importance (15–20%). We then calculated the sample size required to detect this level of reduction with 80% power, and applied an inflation factor to account for the cluster-randomised design. This factor (1·95) was calculated as

$$1+\rho \times \theta -1+\theta \gamma^{2}$$

where ρ is the intraclass correlation for preterm birth, θ is the mean cluster size (assuming equal cluster sizes), and γ is the coefficient of variation in cluster size (which was estimated from our previous study in this area).^39^ We estimated that 3367 livebirths would be required in each group. Because the number of available clusters was fixed, we used previous information about population size and crude birth rates to estimate the time required to reach this sample size.

We assessed the extent to which the randomisation procedure achieved balance across a range of parental, household, and sociodemographic variables. For women in the intervention group, we calculated the proportion of all women providing initial adequate vaginal and urine specimens, the proportion with infections, and the proportions receiving and completing antibiotic regimens. An episode was classified as resolved or persistent on the basis of available follow-up test-of-cure results. We estimated the resolution rate as the proportion of participants who provided a negative test-of-cure sample (ie, subsequent normal Nugent score or non-infected urine specimen) among the number of participants who were infected at the first screening. This analysis was repeated for the 10% control subsample.

For analysis of the effect of the intervention on primary and secondary outcomes, we excluded the 10% control subsample, because the purpose of this group was to provide a comparison of population-level infection rates between study groups. Our main analysis then followed an intention-to-treat approach. The percentage of preterm births was described among livebirths, and the intervention effect size was calculated. We then expanded the pool of analysable pregnancies to include all birth outcomes after more than 20 weeks' gestation, and examined the group-specific incidence of preterm birth outcomes. All effect sizes were estimated with binomial regression models with a log-link function, without covariate adjustment (although we retained an option to adjust for variables if imbalanced between the groups). 95% CIs for intervention effect sizes were calculated from SE estimates adjusted for the cluster randomisation with generalised estimating equations.^39^ We also did a cluster-level analysis by using a *t* test on the cluster-level medians of gestational age to account for skewed gestational age distribution and the non-independence of outcomes within clusters. Among women screened, we additionally estimated rates of preterm delivery for infected women, stratified by treatment and cure status, and compared them with rates in non-infected women. These observational analyses were adjusted for factors known to be associated with abnormal vaginal flora.

We did interim analyses after 33% and 66% of the intended sample had study outcomes. At these interim timepoints, nominal p values less than 0·0007 and 0·16 were deemed significant and were used by the data and safety monitoring board, in consideration of other factors, to recommend early termination of the trial. All analyses were done in Stata (version 14.1). The trial is registered with ClinicalTrials.gov, number NCT01572532.

### Role of the funding source

The study funders had no role in study design, data collection, analysis, or interpretation; or writing of the report. ACL, LCM, SD, and AHB had full access to all the data in the study; AHB had final responsibility for the decision to submit for publication.

## Results

Among 19 455 women under pregnancy surveillance in the study areas, 9952 lived in intervention clusters and 9503 in control clusters. Between Jan 2, 2012, and July 28, 2015, 5127 pregnancies were identified in the intervention clusters and 5185 in the control clusters, with 4840 and 4872 enrolled in each group, respectively (figure). In the control group, 481 pregnancies (10%) were randomly assigned to receive the intervention and excluded from the primary analysis. In the intervention clusters, 104 pregnancies were lost to follow-up; thus, outcome information was available for 4736 pregnancies. Among these pregnancies, 89 resulted in induced abortion and 575 in early miscarriage, and thus 4072 pregnancies (4089 births) were available for analysis (3818 livebirths, 215 stillbirths, and 56 late miscarriages). In the control clusters, 121 pregnancies were lost to follow-up, which meant that outcome information was available for 4270 pregnancies. 94 of these pregnancies resulted in induced abortion, and 416 in early miscarriage, and thus 3760 pregnancies (3783 births) were available for analysis of outcomes (3557 livebirths, 166 stillbirths, and 60 late miscarriages). The frequency of, and reasons for, exclusions and losses to follow-up were similar between study groups. Baseline household and maternal characteristics were similar between groups (table 1). Women in the intervention and control clusters did not differ in age, mean gestational age at enrolment, education, parity, wealth, history of previous neonatal death, mid-upper-arm circumference, or tobacco use (table 1), but the frequency of skilled delivery attendance, the proportion of participants enrolled after the first trimester (data not shown), and frequency of betel nut use were slightly higher in control clusters than in intervention clusters (table 1).

**Figure:**
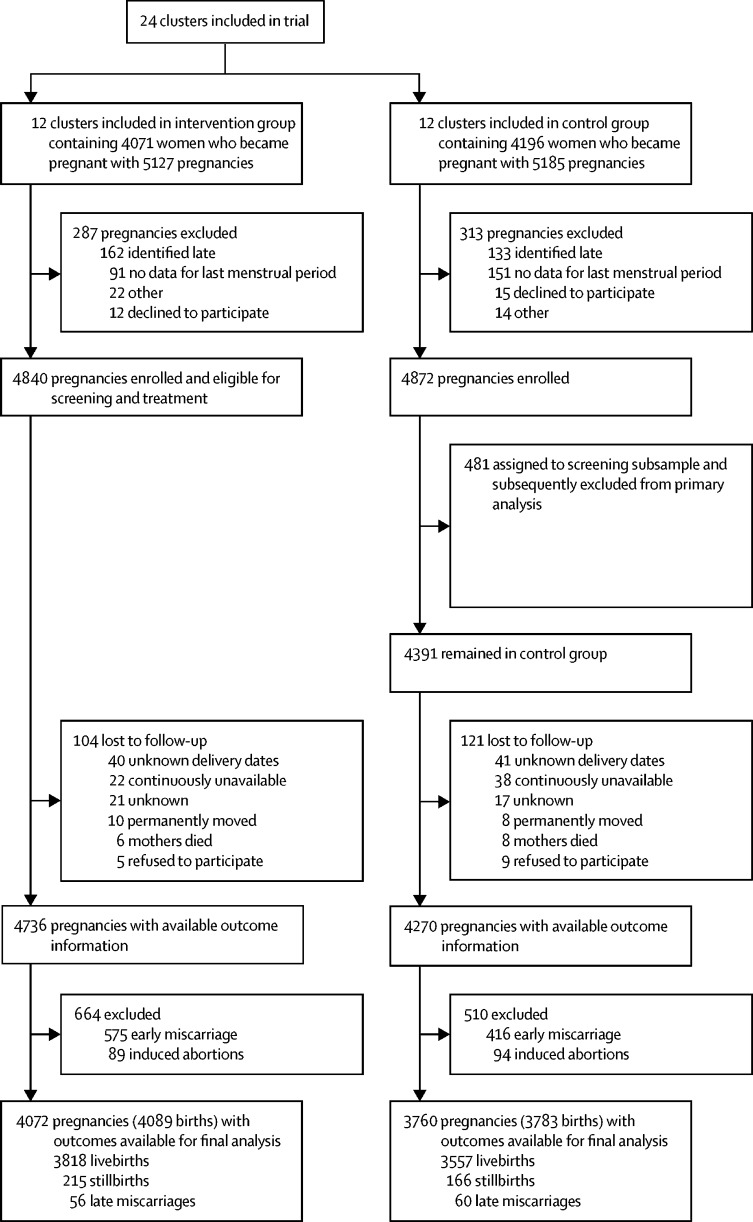
Trial profile

**Table 1:** Baseline maternal, household, and pregnancy characteristics by study group

		**Intervention clusters**	**Control subsample (10% receiving intervention)**	**Control clusters**
**All pregnancies enrolled**				
N		4840	481	4391
Maternal age, years		26·7 (6·1)	27·2 (5·8)	27·5 (6·1)
Gestational age, weeks		10·0 (4·7)	10·0 (3·5)	11·1 (4·1)
Maternal education (completed)				
	None	970 (20%)	77 (16%)	720 (18%)
	Primary	1830 (38%)	184 (38%)	1399 (35%)
	Secondary	1851 (38%)	195 (41%)	1692 (43%)
	Higher	185 (4%)	25 (5%)	157 (4%)
Paternal education (completed)				
	None	1664 (34%)	143 (30%)	1175 (30%)
	Primary	1885 (39%)	191 (40%)	1602 (40%)
	Secondary	1017 (21%)	110 (23%)	932 (23%)
	Higher	270 (6%)	37 (8%)	259 (7%)
Parity		1·6 (1·8)	1·6 (2·2)	1·6 (1·8)
Household wealth quintile				
	1 (poorest)	993 (21%)	87 (18%)	783 (20%)
	2	1018 (21%)	108 (22%)	755 (19%)
	3	956 (20%)	89 (19%)	787 (20%)
	4	983 (20%)	91 (19%)	786 (20%)
	5 (wealthiest)	886 (18%)	106 (22%)	865 (22%)
Antenatal care sought in health system		1736 (43%)	198 (48%)	1752 (47%)
Antenatal care from any provider in health system (≥four visits)		318 (8%)	42 (10%)	348 (9%)
History of previous neonatal death*		375 (12%)	38 (12%)	270 (11%)
Mid-upper-arm circumference, cm		23·7 (2·3)	23·8 (2·7)	23·7 (2·8)
Betel nut use		1971 (48%)	251 (61%)	2396 (65%)
History of chewing tobacco products in pregnancy		324 (8%)	52 (13%)	550 (15%)
Median interbirth interval (IQR), months*		35·6 (24·5–52·9)	34·3 (23·6–55·4)	36·3 (25·3–53·4)
**Livebirths with known pregnancy outcomes**				
N		3818	374	3557
Location of delivery				
	Home	2770 (85%)	269 (82%)	2521 (82%)
	Facility	494 (15%)	59 (18%)	569 (18%)
Skilled assistance at delivery		635 (20%)	86 (26%)	784 (25%)
Caesarean section		241 (7%)	28 (9%)	276 (8%)
Single or multiple birth				
	Single	3787 (99%)	368 (98%)	3520 (99%)
	Twin	28 (1%)	6 (2%)	31 (1%)
	Triplet	3 (<1%)	0 (0%)	6 (<1%)
Infant sex				
	Female	1874 (50%)	160 (43%)	1727 (49%)
	Male	1908 (50%)	213 (57%)	1797 (51%)

Data are mean (SD) or n (%), unless otherwise specified. Missing data for different variables are detailed in the appendix.

* Only participants who reported one or more previous livebirths were asked this question.

578 pregnancies in the intervention group and 50 in the control subsample ended before the specimen collection visit, and thus 4262 and 431 pregnancies, respectively, were eligible for infection screening, (table 2). 3817 (90%) of 4262 participants in the intervention group provided adequate-quality vaginal specimens, and 3668 (86%) provided adequate-quality urine samples. In the control subsample, 384 (89%) of 431 participants provided adequate-quality vaginal specimens, and 366 (85%) provided adequate-quality urine samples. In the initial screening, 622 of 3817 participants in the intervention group and 72 of 384 in the control subsample had abnormal vaginal flora (table 2), corresponding to prevalences of 16·3% (95% CI 15·1–17·6) and 18·8% (14·3–24·6), respectively. Among women diagnosed with abnormal vaginal flora, 86% of women in the intervention clusters were started on clindamycin and 77% completed the full course (table 2). At rescreening, samples were collected from 472 (76%) of the 622 participants diagnosed with abnormal vaginal flora in the intervention group (table 2). 157 (33%) of 472 participants had persistent abnormal vaginal flora, whereas 315 (67%) were uninfected, irrespective of treatment status (table 2). Among the 423 participants who completed the full antibiotic course and underwent rescreening, 286 (68%) were cured. 142 (90%) of the 157 participants with persistent abnormal vaginal flora were treated a second time and 124 (79%) completed the full antibiotic course (table 2). Overall, the effective coverage of documented treatment success with repeated screening for, and treatment of, abnormal vaginal flora was 58% in the intervention group (table 2). In the control subsample, treatment and resolution frequencies were broadly similar (table 2). Minor adverse events reported from antibiotic use are reported in the appendix.

**Table 2:** Screening of, and treatment for, AVF and UTI

			**Intervention cluster pregnancies**	**Control subsample pregnancies**
First AVF screening				
	Eligible for screening*		4262	431
	Adequate screening specimen collected		3817/4262 (90%)	384/431 (89%)
	Positive for AVF		622/3817 (16%)	72/384 (19%)
		Treatment started	536/622 (86%)	59/72 (82%)
		Treatment completed	477/622 (77%)	52/72 (72%)
Second AVF screening				
	Adequate screening specimen collected		472/622 (76%)	47/72 (65%)
	AVF resolution		315/472 (67%)	30/47 (64%)
	Positive for AVF		157/472 (33%)	17/47 (36%)
		Treatment started	142/157 (90%)	14/17 (82%)
		Treatment completed	124/157 (79%)	12/17 (71%)
Third AVF screening				
	Adequate screening specimen collected		119/157 (76%)	10/17 (59%)
	AVF resolution		46/119 (39%)	4/10 (40%)
	Positive for AVF		73/119 (61%)	6/10 (60%)
	Overall AVF resolution		361/622 (58%)	34/72 (47%)
First urine screening				
	Adequate screening specimen collected		3668/4262 (86%)	366/431 (85%)
	Positive for UTI		317/3668 (9%)	43/366 (12%)
		Treatment started	271/317 (85%)	37/43 (86%)
		Treatment completed	251/317 (79%)	33/43 (77%)
Second urine screening				
	Adequate screening specimen collected		244/317 (77%)	32/43 (74%)
	Resolved UTI		197/244 (81%)	23/32 (72%)
	Persistent UTI		47/244 (19%)	9/32 (28%)
		Second treatment started for persistent UTI	37/47 (79%)	8/9 (89%)
		Second treatment completed for persistent UTI	33/47 (70%)	7/9 (78%)
Third urine screening				
	Adequate screening specimen collected		41/47 (87%)	6/9 (67%)
	Urine clearance (negative culture)		31/41 (76%)	4/6 (67%)
	Persistent infection		10/41 (24%)	2/6 (33%)
Overall UTI resolution			224/317 (71%)	27/43 (63%)

AVF=abnormal vaginal flora. UTI=urinary tract infection.

* 578 of 4840 pregnancies in the intervention group and 50 of 481 pregnancies in the control subsample ended before the specimen collection visit.

317 of 3668 participants in the intervention group, and 43 of 366 in the control subsample, had a urinary tract infection at initial screening (table 2), corresponding to prevalences of 8·6% (95% CI 7·7–9·5) and 11·8% (8·6–15·5), respectively. In the intervention group, 271 (85%) participants with urinary tract infections started antibiotics and 251 (79%) completed the full course (table 2). A test-of-cure urine specimen was obtained from 244 (77%), 47 (19%) of whom had persistent infections (table 2). Among the 216 participants who completed the full initial antibiotic course and underwent rescreening, 153 (71%) were cured. Overall, the effective coverage of successful treatment of urinary tract infection was 70·7% after two antibiotic courses (table 2). The frequency of treatment and resolution were broadly similar in the control subsample. At initial screening, 73 (2%) of 3319 participants in the intervention group were co-infected with abnormal vaginal flora and urinary tract infections.

The distribution of mean gestational age was similar between the intervention (38·7 weeks [SD 3·1]) and control (38·9 weeks [3·1]) groups (mean difference −0·14 [95% CI −0·37 to 0·07]). A cluster-level analysis of median gestational age similarly showed no difference between groups (data not shown). The incidence of preterm livebirths of less than 37 weeks' gestation (21·8% in the intervention group *vs* 20·6% in the control group; RR 1·07 [95% CI 0·91–1·24]; coefficient of variation k=0·166), preterm livebirths of less than 34 weeks' gestation (7·2% *vs* 7·3%; 1·00 [0·81–1·24]), or preterm deliveries including late miscarriage and stillbirth (23·6% *vs* 22·7%; 1·04 [0·90–1·21]) did not differ significantly between groups (table 3). Sensitivity analysis showed that inclusion of birth outcomes for pregnancies of less than 20 weeks' gestation did not affect outcomes (data not shown). Adjustment for covariates (table 1) that seemed slightly imbalanced between groups also did not affect these estimates (data not shown).

**Table 3:** Effect of screening for, and treatment of, maternal abnormal vaginal flora and urinary tract infection on primary and secondary outcomes

		**Intervention group**	**Control group**	**Relative risk (95% CI)**
Pregnant women with outcome data		4736	4270	..
Birth outcomes for fetuses >20 weeks*		4089	3783	..
	Livebirths	3818	3557	..
	Stillbirths	215	166	..
	Late miscarriage	56	60	..
Preterm livebirths <37 weeks (primary outcome)		834/3818 (21·8%)	731/3557 (20·6%)	1·07 (0·91–1·24)
Secondary outcomes				
	Preterm livebirths <34 weeks	276/3818 (7·2%)	258/3557 (7·3%)	1·00 (0·81–1·24)
	All preterm outcomes (preterm livebirths and stillbirths, late miscarriage)	963/4089 (23·6%)	859/3783 (22·7%)	1·04 (0·90–1·21)
	Late miscarriage (20–27 weeks)	56/4089 (13·7/1000)	60/3783 (15·9/1000)	0·88 (0·54–1·45)
	Late fetal deaths (>20 weeks)	271/4089 (66·3/1000)	226/3783 (59·7/1000)	1·11 (0·85–1·44)
	Stillbirth (≥28 weeks)	215/4033 (53·3/1000)	166/3723 (44·6/1000)	1·19 (0·91–1·55)
	Neonatal mortality rate	120/3818 (31·4/1000)	134/3557 (37·7/1000)	0·82 (0·57–1·18)
	Perinatal death (stillbirths plus neonatal deaths before age 7 days)	316/4033 (78·4/1000)	274/3723 (73·6/1000)	1·05 (0·80–1·37)
	Low birthweight†	543/2461 (22·1%)	498/2268 (22·0%)	1·00 (0·73–1·35)
	Small for gestational age‡	829/2341 (35·4%)	881/2138 (41·2%)	0·86 (0·74–1·01)
	Maternal clinical urinary tract infection (>20 weeks)§	290/3809 (7·6%)	340/3533 (9·6%)	0·83 (0·36–1·90)
	Maternal clinical pyelonephritis (>20 weeks)§	10/3809 (0·3%)	14/3553 (0·4%)	0·69 (0·23–2·08)

Data are N, n/N (%), or n/N (rate), unless otherwise specified. Primary and secondary outcomes are defined in the appendix.

* Includes multiple births.

† Infants were weighed within the first 72 h of life; reasons for missing or late data include death, missed postnatal visits, caretaker refusal, and loss to follow-up (ie, unable to contact family).

‡ Infants whose birthweights were <10% birthweight cutoff for gestational age and sex as defined by the Intergrowth-21st neonatal birthweight standards.^45^

§ Assessed only among pregnancies resulting in one or more livebirths, stillbirths, or late miscarriages.

In exploratory post-hoc analyses (appendix), the risk of preterm delivery was significantly higher among women and girls with persistent abnormal vaginal flora than among non-infected participants (adjusted RR [adjusted for age, wealth index, and primiparity] 1·45 [95% CI 1·20–1·76]). Preterm delivery (ie, before 37 weeks) occurred in 72 (36%) of 202 participants with persistent abnormal vaginal flora, compared with 839 (24%) of 3472 non-infected participants. The frequency of delivery before 34 weeks' gestation was also higher among those with persistent abnormal vaginal flora than among those who were not infected (19·3% *vs* 10·2%; adjusted RR 1·88 [95% CI 1·41–2·54]). In participants who were diagnosed with abnormal vaginal flora, completed antibiotic treatment, and had documented cure, the risks of preterm birth before 37 weeks' (83 [21%] of 387) and 34 weeks' (29 [7%] of 387) gestation was similar to that in uninfected participants (appendix).

Rates of late miscarriage, late fetal deaths, stillbirth, neonatal mortality, and perinatal mortality did not differ between groups (table 3). Infant weight was measured within 72 h of birth for 2461 (64%) of 3818 infants in the intervention group and 2268 (64%) of 3557 infants in the control group. Mean weight did not differ significantly between groups (2800 g [SD 516] in the intervention group *vs* 2779 g [467] in the control group). The frequency of infants with low birthweight or who were small for gestational age did not differ significantly between groups (table 3).

## Discussion

In rural Sylhet, Bangladesh, a population-based screening and treatment intervention for maternal genitourinary tract infections in early pregnancy had no effect on the incidence of preterm livebirths, or other pregnancy outcomes. Although we initiated antibiotic treatment in most infected women and attempted to retreat persistent infections, the overall effective coverage of successful treatment was low (58% for abnormal vaginal flora and 71% for urinary tract infections). The lack of intervention effect was probably caused by the low rates of effective treatment coverage and clinical cure achieved for both infections. Antibiotic resistance and potential variation in the microbial composition of abnormal vaginal flora and host immune responses in our population could have contributed to this lack of response. Although our study had a null effect, our findings are important because they highlight the need to better describe the local microbiology of these infections, to identify treatment with improved clinical efficacy, and to develop alternative strategies to prevent preterm births in LMICs.

The prevalence of urinary tract infection in our population-based screening was similar to those in other reports from rural Bangladesh. In Rajshahi district, asymptomatic bacteriuria affected 4–12% of mothers presenting to antenatal clinics.^46^, ^47^ To our knowledge, there are no other reports of the prevalence of abnormal vaginal flora in Bangladesh. However the previously published^48^ prevalence of bacterial vaginosis in our study (9·8%) was similar to that in other studies in Bangladesh. In Ghaibandha, the prevalence of bacterial vaginosis was 7·6%.^41^ In urban Bangladeshi populations, the prevalence of bacterial vaginosis has been reported to be 17·7–59·5%.^49^, ^50^, ^51^, ^52^, ^53^, ^54^

The quality of evidence for the effect of antibiotic treatment for asymptomatic bacteriuria in pregnancy on preterm birth and low birthweight was graded as low in a 2015 Cochrane review.^55^ Two studies^56^, ^57^ showed a reduction in preterm birth with treatment of asymptomatic bacteriuria (RR 0·27 [95% CI 0·11–0·62]; n=242 participants). However, study quality was low and the interventions were heterogeneous. In one, a quasi-experimental randomised controlled trial^56^ published in 1969, mothers were treated with continuous antibiotics until delivery, whereas the other was a placebo-controlled randomised controlled trial of treatment of asymptomatic group B streptococcus bacteriuria.^57^ Pooled analysis of six studies dating from 1960–75 with 1437 participants overall showed a reduction in low birthweight (RR 0·64 [95% CI 0·45–0·93]).^55^ Again, study quality was graded as low, and interventions differed, with continuous daily antibiotics provided in four of the studies.

In our trial, treatment of asymptomatic bacteriuria and urinary tract infections had no effect on preterm birth or low birthweight. We did not provide continuous antibiotics as per the previous trials, but provided a short antibiotic course and repeat treatment for persistent infections based on antibiotic sensitivity. The rate of antibiotic resistance was high among uropathogens in our study, which necessitated a change in the antibiotic regimen mid-study (these results will be reported elsewhere). The WHO Global Surveillance of Antimicrobial Resistance reported high rates of resistance in *Escherichia coli* (16–68% resistance to third-generation cephalosporins and 32–64% resistance to fluoroquinolones) in national data from five countries in southeast Asia.^58^, ^59^ Safety of antibiotic regimens is a consideration in pregnancy and limits the choice of therapeutic, bactericidal antimicrobials. Antibiotic stewardship and development of effective antimicrobials are crucial priorities to improve the efficacy of treatment of urinary tract infections during pregnancy in LMICs.^59^, ^60^

A Cochrane review^20^ of data from 13 trials of bacterial vaginosis (combined n=6491) concluded that treatment of asymptomatic bacterial vaginosis in the general obstetric population did not reduce the risk of preterm birth (pooled RR 0·88 [95% CI 0·71–1·09]). One of the largest studies, the National Institute of Child Health and Human Development's Maternal Fetal Medicine Unit trial,^61^ showed that treatment of bacterial vaginosis with metronidazole did not affect preterm delivery in low-risk obstetric populations. Furthermore, mothers with asymptomatic trichomonas who received metronidazole had increased rates of preterm birth compared with those who received placebo.^61^ Results from the PREMEVA1 trial^62^ published in 2018 showed that treatment of bacterial vaginosis in early pregnancy with oral clindamycin did not reduce rates of late abortion or spontaneous preterm birth.

However, reductions in the incidence of preterm birth were reported in previous trials that targeted abnormal vaginal flora (ie, intermediate flora in addition to bacterial vaginosis). In a pooled analysis in the Cochrane review^20^ of two trials^22^, ^23^ (combined n=894) that targeted abnormal vaginal flora (ie, intermediate flora and bacterial vaginosis), treatment of abnormal vaginal flora was associated with significant reductions in the frequency of preterm birth (ie, <37 weeks' gestation; RR 0·53 [95% CI 0·34–0·84]).

Unlike in these two trials,^22^, ^23^ in which individual participants were randomly assigned to receive either clindamycin or placebo, we assessed a population-level screening and treatment approach. The overall effective coverage of treatment success for abnormal vaginal flora in our trial was low (58%) despite repeated oral clindamycin therapy for persistent infection. In our post-hoc analysis, compared with participants without abnormal vaginal flora, persistent abnormal vaginal flora was associated with a 45% increased risk of preterm birth, even after adjustment for other risk factors. Conversely, participants with abnormal vaginal flora who responded to antibiotic therapy had risks of preterm birth similar to those of their non-infected peers. Although we did not identify other studies in which the prevalence of persistent abnormal vaginal flora was reported, persistent bacterial vaginosis is a well described clinical entity, with a frequency of recurrence of 15–30% within 3 months of metronidazole treatment and only half of mothers remaining non-infected in long-term follow-up.^63^, ^64^, ^65^ We anticipated higher rates of cure with oral clindamycin in our trial, because the drug has good clinical efficacy against intermediate flora, anaerobic species,^66^ and persistent bacterial vaginosis^67^ (specifically metronidazole-resistant *Gardnerella vaginalis*).^68^ The microbial composition of abnormal vaginal flora could differ in the Bangladeshi population compared with that in the UK study populations in which the clinical efficacy of clindamycin was established.^22^, ^69^ Abnormal vaginal flora is a heterogeneous, polymicrobial condition, and the vaginal microbiome varies between ethnic groups in the USA and Africa.^70^, ^71^, ^72^ Differences in antibiotic response have also been reported between US and Kenyan women.^73^ Furthermore, certain microbiota, including bacterial-vaginosis-associated bacteria-2^74^ and *Lactobacillus iners*,^75^ are associated with persistent bacterial vaginosis and vaginal inflammation. Study of the vaginal microbiome in our population is needed to identify the specific microbiota associated with persistent abnormal vaginal flora and to target diagnostics and antimicrobial treatment against these species.

Beyond the identification of microbiota associated with abnormal vaginal flora in this population, it is crucial to understand the role of these microbiota in host immune responses to elucidate the pathophysiology of preterm birth.^71^ Characterisation of the host inflammatory response to microbiota associated with abnormal vaginal flora may help to clarify why the intervention had no effect in our population. For example, single nucleotide polymorphisms in pro-inflammatory cytokines (eg, tumour necrosis factor α, interleukins 6 and 1β) are associated with preterm birth,^76^, ^77^ and several investigators have reported a gene–environment interaction in which bacterial vaginosis could modify the host inflammatory response.^78^, ^79^, ^80^

Our trial differed in terms of methods and population from previous trials of treatment of abnormal vaginal flora^22^, ^23^ and lower genital tract screening,^81^ in which effects on preterm birth were reported. Our study population was primarily a high-risk rural population with low engagement in antenatal care and low rates of institutional delivery. Previous studies were done in high-income UK^22^, ^23^ and Austrian^81^ populations recruited from referral hospitals and antenatal care clinics. We randomly assigned population clusters to receive the screening and treatment intervention, whereas in previous studies^22^, ^23^ individual mothers were randomly assigned. In the Austrian study,^81^ participants were additionally screened and treated for trichomonas and candida infections. Other explanations for the absence of an effect in our trial include potential baseline differences or unobserved heterogeneity between study groups. Finally, there simply might not have been any treatment effect.

Our study had several limitations. We tested for infections before 20 weeks' gestation, because we hypothesised that early treatment would prevent bacterial seeding of the amniotic fluid. We did not test for infection in later pregnancy. We targeted two infections that we hypothesised would have high prevalence in our population. However, we did not screen for other genital tract infections, such as trichomonas, candida, or sexually transmitted infections that have been associated with preterm birth for which treatment has been effective.^81^, ^82^ Another limitation is that our study findings might not be generalisable to other regions. Infection prevalence was low in our rural south Asian population. Bacterial vaginosis affected 38% of pregnant women in a study in South Africa and Kenya,^83^ and the prevalence of asymptomatic bacteriuria was 86·6% in a Nigerian study.^25^ A screening and treatment programme might have more of an effect on the incidence of preterm birth in settings with higher infection burden. We did not have clinical data of sufficient quality to disaggregate preterm birth as spontaneous versus medically indicated. However, most preterm births are spontaneous in this setting, because access to intrapartum care is poor. Only 6% of preterm births were delivered by caesarean section in our population. Finally, our method of establishing gestational age could be considered a limitation. Ultrasonography was not feasible in this trial. We used a menstrual calendar with monthly pregnancy surveillance for early detection of missed periods. This method was validated in Ghaibandha, Bangladesh,^44^ and had high diagnostic accuracy for identification of preterm births compared with ultrasonographic dating (sensitivity 96%, specificity 89%).^44^ Thus, we think that our gestational age estimates were accurate for measurement of the primary study outcome.

Our findings highlight the need for further research to improve data for the epidemiology and microbiology of abnormal vaginal flora and urinary tract infections in the south Asian population. Characterisation of the vaginal microbiota of abnormal vaginal flora—and particularly of persistent abnormal vaginal flora—is crucial to identify the specific subtypes associated with preterm birth and to help to better target screening tools and develop treatments with improved clinical efficacy. The high rate of antibiotic resistance emphasises the urgent need for antibiotic stewardship in LMICs and development of new antibiotics that are safe to use in pregnancy. Finally, additional research is needed to study the role of other infections and risk factors for preterm birth that we did not consider in our intervention. In low-income settings, comprehensive approaches to improve the quality of antenatal care and targeting of known risk factors still hold promise, but will require concerted, innovative research and public health efforts to prevent the large burden of preterm-birth-related morbidity and mortality globally.
