# Supplementary material for: Effect of population-based antenatal screening and treatment of genitourinary tract infections on birth outcomes in Sylhet, Bangladesh (MIST): a cluster-randomised clinical trial
Source: Lancet Glob Health. 2018 Dec 13;7(1):e148–59. doi: 10.1016/S2214-109X(18)30441-8 (PMC6293967; doi:10.1016/S2214-109X(18)30441-8)
Supplement: Supplementary appendix [file mmc1.pdf]

THE LANCET
Global Health

Supplementary appendix

This appendix formed part of the original submission and has been peer reviewed. We post it as supplied by the authors.

Supplement to: Lee ACC, Mullany LC, Quaiyum M, et al. Effect of population-based antenatal screening and treatment of genitourinary tract infections on birth outcomes in Sylhet, Bangladesh (MIST): a cluster-randomised clinical trial. *Lancet Glob Health* 2019; **7:** e148–59.

Web Appendix

For "The impact of a population-based antenatal screening and treatment program for genitourinary tract infections on birth outcomes in Sylhet, Bangladesh: a cluster randomized clinical trial"

Anne CC Lee; Luke C Mullany; Mohammad Quaiyum; Dipak K Mitra; Alain Labrique; Parul Christian; Parvez Ahmed; Jamal Uddin; Iftekhar Rafiqullah; Sushil DasGupta; Mahmoodur Rahman; Emilia H Koumans; Salahuddin Ahmed; Samir K. Saha; and Abdullah H. Baqui for the Projahnmo Study Group in Bangladesh

Table of Contents

eFigure 1. Map of Projahnmo MIST Study Area, Sylhet, Bangladesh……………………..……………………2

eTable 1. Definitions of Study Outcomes…………………………………………………..………………3

eMethods. Genitourinary Tract Infection Screening and Treatment ……..…………………….……..…………4

eResults: Adverse Events Associated With Intervention……..…………….……………………..…..…………5

eTable 2. Table 1: Baseline maternal, household, and pregnancy characteristics by study group…….………6

eTable 3. Maternal Abnormal Vaginal Flora Status and Preterm Delivery Risk…….………………….....……8

Appendix References……………………………………..……………………………………..…..…………9

eFigure 1. Map of Projahnmo MIST Study Area, Sylhet, Bangladesh

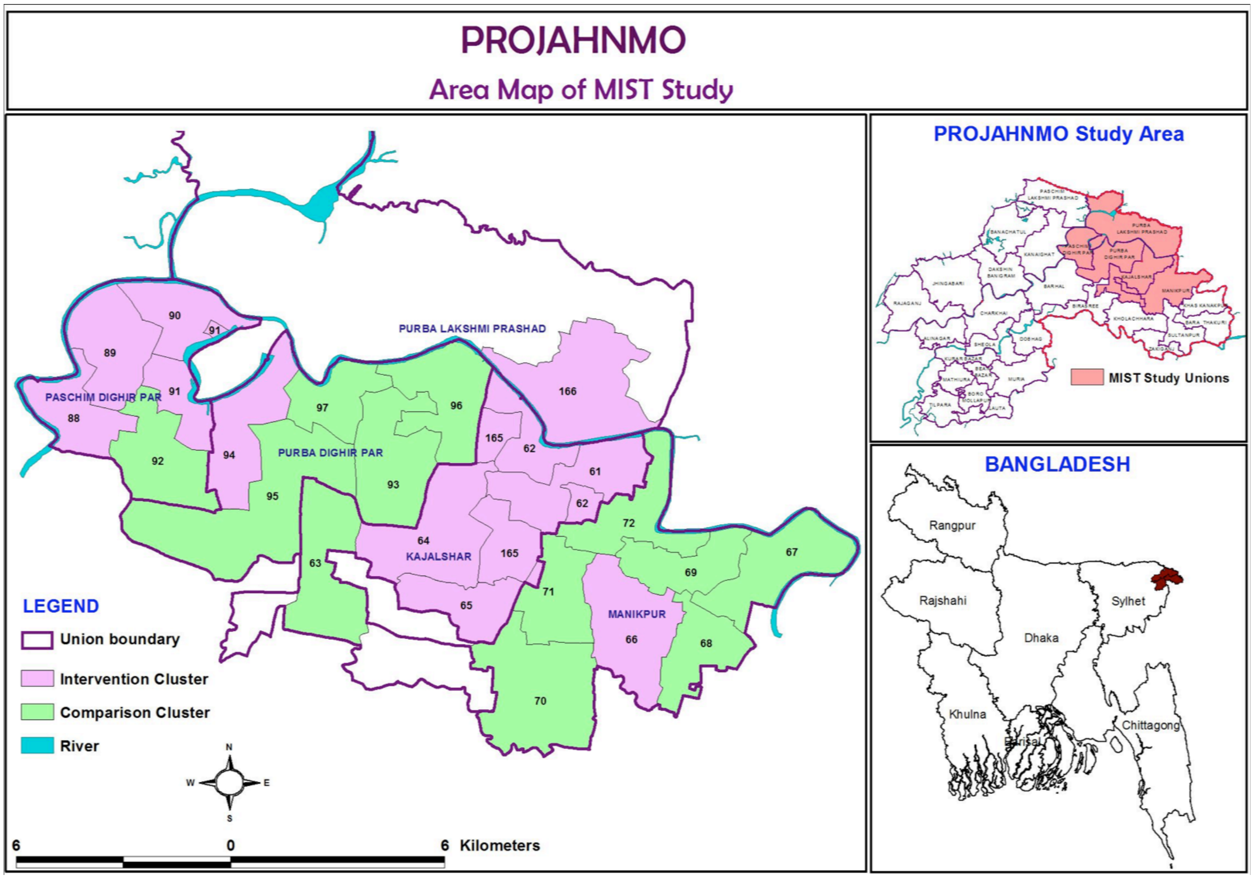

eTable 1. Definitions of Study Outcomes

	Numerator	Denominator
Primary outcome		
Preterm live birth	Live births <37 weeks of gestation	All live births
Secondary outcomes		
Preterm birth and late miscarriage	• Live births (28-<37weeks)<br>• Stillbirth (28-<37 weeks)<br>• Late Miscarriage (20-<28 weeks)	All birth outcomes ≥20 weeks
Late miscarriage rate	All spontaneous abortion/miscarriage (non-therapeutic abortions) occurring between 20-<28 weeks gestation	All birth outcomes ≥20 weeks
Stillbirth rate	Stillbirth (≥28 weeks gestation) and no signs of life	All live births and stillbirths ≥28 weeks
Late fetal death rate	All late miscarriages and stillbirths occurring >20 weeks gestation	All birth outcomes ≥20 weeks
Perinatal mortality rate	Stillbirth AND early neonatal death (≥7 days of life)	All live births and stillbirths ≥28 weeks
Neonatal mortality	All neonatal death (≥28 days of life)	All live births
Low Birthweight	Infants with birthweight (weight measured within 72 hours of birth) <2500 gm	All live births with birthweight measured
WHO[a]-defined possible serious bacterial infection[1]	In the first 28 days of life:<br>• not able to feed since birth or stopped feeding well (confirmed by observation)<br>• convulsions<br>• fast breathing (60 breaths per minute or more) among infants less than 7 days old<br>• severe chest in-drawing<br>• no movement, or movement only upon stimulation<br>• fever (≥38 °C or greater) or low body temperature (<35.5 °C)	All live births
Maternal Morbidity (puerperal sepsis)	In first 10 days post birth:<br>• High fever (>39.0 °C) at home visit<br>• Fever (38.0-39.0 °C) or history of fever paired with at least one additional symptom of sepsis (lower abdominal pain, pelvic pain, or abnormal/foul-smelling discharge)	All pregnancies
Maternal clinical UTI[a]	At any pregnancy ANC[a] visit post 20 weeks, or post-partum visit:<br>Dysuria, urinary frequency, urinary pressure, abdominal pain at a subsequent visit	All pregnancies
Maternal clinical pyelonephritis	At any post 20 week pregnancy ANC visit or post partum visit: Maternal fever AND flank/back pain	All pregnancies
SGA[a] (Intergrowth)	<10% birth weight for GA by sex compared to Intergrowth reference[2]	All live births

[a]Abbreviations: WHO, World Health Organization; UTI, urinary tract infection; ANC, antenatal care; SGA, small-for-gestational-age; GA, gestational age

eMethods

GU Infection Screening

AVF: Self-administered vaginal swabs were collected and rolled onto a plain glass slide in the field by CHWs. In the lab, slides were gram stained and graded by Nugent scoring,[3] assessing for relative concentrations of lactobacillus, gardnerella/bacteroides, and curved gram-variable rods. Microbiologists were scored [3] by microbiologists trained and standardized by a study investigator prior to the start of the study (AL). BV was defined as Nugent scores 7-10, intermediate flora as 4-6, and AVF as $\geq$ 4.[4] During the study, a random sample of 5% of study slides were reviewed by the study investigator (AL) for quality control purposes.

UTI: A clean catch midstream urine specimen was collected and urine culture performed in the Sylhet field laboratory. Urine cultures were performed based on standard microbiology laboratory technique. CLSI standard protocol was followed for antibiotic sensitivity test. Microbiologists were trained at WHO reference laboratory at Dhaka Shishu Hospital to perform and document urine culture results as per study protocol. A random 5% of the urine isolates were sent to Dhaka and confirmed at this Dhaka Shishu reference laboratory for quality assurance.
Bacterial growth was classified as 1) high-burden: $>10^5$ colony forming units (CFU)/1mL of urine of a single uropathogen,[5] or 2) intermediate growth: $>10^3$-10^5 CFU/mL of a single uropathogen. At the time of the specimen collection, CHWs inquired about symptoms of UTI (dysuria, urinary frequency, hematuria, abdominal pain, fever, flank pain).

GU Infection Treatment

Detailed treatment algorithms and protocols are detailed in our protocol paper.[6] Women with clinical symptoms were referred to the sub-district hospital for evaluation.[6] CHWs distributed antibiotics to infected women at home, and village health workers monitored for adverse effects.[6] AVF was treated with oral clindamycin 300 mg twice daily for 5 days.[7] Three weeks later, women were rescreened and retreated as necessary. A final vaginal sample was collected 3 weeks after the second course of antibiotics, and women who failed to respond to the second treatment were referred to Sylhet Osmani Medical College Hospital. For UTI, the antibiotic treatment was initially Cefixime. In October 2012, after high rates of Cefixime resistance were uncovered, the antibiotic was changed to oral Macrobid/Nitrofurantoin 100 mg twice daily for 7 days. For positive urine cultures, a repeat urine culture was obtained one week after completion of antibiotics. If the second urine culture was positive, the supervising field physician selected an appropriate antibiotic based on the prior culture's antimicrobial sensitivity pattern. Persistent UTI was referred to Sylhet Medical College Hospital for evaluation and management.

eResults: Adverse Events Associated with Intervention

We did not receive any report of severe or unanticipated adverse events associated with infection screening or antibiotic treatment. There were 36 cases of minor/mild antibiotic associated adverse events including loose stools, nausea, or abdominal cramping (Clindamycin n=35, Cefixime n=1). Among these cases there was 1 report of a minor rash that was noted after antibiotic treatment. There were no reported cases of bloody or mucoid stools, or other symptoms that may have been consistent with pseudomembranous colitis. All mild adverse events were locally managed with rice-based oral rehydration solution, counseling, and resolved upon close follow up by our field medical officers.

Table 1. Baseline maternal, household, and pregnancy characteristics by study arm

	Intervention Clusters	Control sub-sample (10% receiving intervention)	Control Clusters
**Characteristics of all Pregnancies Enrolled**			
Number of Pregnancies (N)	4840	481	4391
Maternal Age at enrollment [years; mean(SD)][a]	26·7 (6·1)	27·2 (5·8)	27·5 (6·1)
Gestational Age at enrollment[a] [weeks; mean(SD)]	10·0 (4·7)	10·0 (3·5)	11·1 (4·1)
Maternal Education (completed)[b] [N(%)]			
None	970 (20%)	77 (16%)	720 (18%)
Primary	1830 (38%)	184 (38%)	1399 (35%)
Secondary	1851 (38%)	195 (41%)	1692 (43%)
Higher	185 (4%)	25 (5%)	157 (4%)
Paternal Education (completed)[b] [N(%)]			
None	1664 (34%)	143 (30%)	1175 (30%)
Primary	1885 (39%)	191 (40%)	1602 (40%)
Secondary	1017 (21%)	110 (23%)	932 (24%)
Higher	270 (6%)	37 (8%)	259 (7%)
Parity [Mean(SD)][c]	1·6 (1·8)	1·6 (2·2)	1·6 (1·8)
Household Wealth Quintile[d] [N(%)]			
1st (poorest)	993 (21%)	87 (18%)	783 (20%)
2nd	1018 (21%)	108 (23%)	755 (19%)
3rd	956 (20%)	89 (19%)	787 (20%)
4th	983 (20%)	91 (19%)	786 (20%)
5th (wealthiest)	886 (18%)	106 (22%)	865 (22%)
Antenatal Care sought in health system[e] [N(%)]	1736 (43%)	198 (48%)	1752 (48%)
Antenatal Care from any provider in health system ($\geq$4 visits)[e] [N(%)]	318 (8%)	42 (10%)	348 (9%)
History of prior neonatal death[f] [N(%)]	375 (12%)	38(12%)	270 (11%)
Mid-upper arm circumference at enrollment [cm; mean(SD)][g]	23.7 (2.3)	23.8 (2.7)	23.7 (2.8)
Betel nut use[h] [N(%)]	1971 (48%)	251 (61%)	2396 (65%)
History of Chewing Tobacco products in pregnancy[h] [N(%)]	324 (8%)	52 (13%)	550 (15%)
Interbirth interval [months; median (IQR)][i]	35·6 (24·5 – 52·9)	34·3 (23·6 – 55·4)	36·3 (25·3 – 53·4)

Characteristics of Live Births Among Enrolled Women with Known Pregnancy Outcomes			
Known Pregnancy Outcomes	3818	374	3557
Location of delivery[j] [N(%)]<br>- Home<br>- Facility	2770 (85%)<br>494 (15%)	269 (82%)<br>59 (18%)	2521 (82%)<br>569 (18%)
Skilled assistance at delivery[k] [N(%)]	635 (20%)	86 (26%)	784 (25%)
C-section[l] [N(%)]	241 (7%)	28 (9%)	276 (9%)
Singleton or multiple birth [N(%)]<br>- Singleton<br>- Twin<br>- Triplet	3787 (99%)<br>28 (1%)<br>3 (0·1%)	368 (98%)<br>6 (2%)<br>0 (0·0%)	3520 (99%)<br>31 (1%)<br>6 (0·2%)
Infant Sex[m] [N(%)]<br>- Female<br>- Male	1874 (50%)<br>1908 (50%)	160 (43%)<br>213 (57%)	1727 (49%)<br>1797 (51%

Missingness of variables reported in Table 1. Missingness reported as N(%)

[a]Missing Age or Gestational Age: Intervention-0 (0·0%); Control Sub-Sample-0 (0·0%); Control-0 (0·0%)

[b]Missing Education: Intervention-4 (0·08%); Control Sub-Sample-0 (0·0%); Control-423 (9·63%)

[c]Missing Parity: Intervention-16 (0·33%); Control Sub-Sample-1 (0·2%); Control-432 (9·45%)

[d]Missing Health Wealth Quintile: Intervention-4 (0·08%); Control Sub-Sample-0 (0·0%); Control-415 (9·45%)

[e]Missing Antenatal Care: Intervention-767 (15·85%); Control Sub-Sample-67 (13·9%); Control-701 (15·96%)

[f]No missing; only asked among those reporting one or more live births (Intervention-3,218; Control Sub-Sample 312; Control-2,544)

[g]Missing MUAC: Intervention-12 (0·25%); Control Sub-Sample-2 (0·4%); Control-732 (16·67%)

[h]Missing Betelnut or Tobacco: Intervention-764 (15·79%); Control Sub-Sample-68 (14·1%); Control-722 (16·44%)

[i]Missing Interbirth Interval (only asked among those reporting live birth): Intervention-340 (10·5%); Control Sub-Sample-59 (18.8%); Control-502 (19·6%)

[j]Missing Delivery Location: Intervention-554 (14·5%); Control Sub Sample-46 (12·3%); Control-467 (13·1%)

[k]Missing Skilled Assistance: Intervention-580 (15·2%); Control Sub Sample-47 (12·6%); Control-476 (13·4%)

[l]Missing C-section: Intervention-536 (14·04%); Control Sub-Sample-45 (12.0%); Control-445 (12.5%)

[m]Missing Sex: Intervention-36 (1·0%); Control Sub Sample-1 (0·3%); Control-33 (1·0%)

eTable 3. Maternal Abnormal Vaginal Flora Status and Preterm Delivery Risk

	Total	<37 weeks gestation	
	N	N (%)	aRR[a]
AVF Infection Status			
Not infected	3,472	839 (24.2%)	REF
Infected, No recorded treatment	95	22 (32.2%)	1.00 (0.70-1.44)
Infected, treated, and CURED	387	83 (21.5%)	0.84 (0.69-1.03)
Persistent Infection: Treated without cure	202	72 (35.6%)	1.45 (1.20-1.76)
TOTAL	4,156	1,016	

[a]aRR (Adjusted Relative Risk): adjusted for other preterm birth risk factors, including maternal age, wealth index and primiparity.
[b]AVF, abnormal vaginal flora

References (Online-Only Appendix)

1. WHO/UNICEF Joint Statement: Managing possible serious bacterial infection in young infants 0–59 days old when referral is not feasible. Geneva, Switzerland: World Health Organization (WHO);2017.
2. Papageorghiou AT, Ohuma EO, Altman DG, et al. International standards for fetal growth based on serial ultrasound measurements: the Fetal Growth Longitudinal Study of the INTERGROWTH-21st Project. Lancet (London, England). 2014;384(9946):869-879.
3. Nugent RP, Krohn MA, Hillier SL. Reliability of diagnosing bacterial vaginosis is improved by a standardized method of gram stain interpretation. J Clin Microbiol. 1991;29(2):297-301.
4. Donders GG. Definition and classification of abnormal vaginal flora. Best Pract Res Clin Obstet Gynaecol. 2007;21(3):355-373.
5. ACOG Practice Bulletin No. 91: Treatment of urinary tract infections in nonpregnant women. Obstet Gynecol. 2008;111(3):785-794.
6. Lee AC, Quaiyum MA, Mullany LC, et al. Screening and treatment of maternal genitourinary tract infections in early pregnancy to prevent preterm birth in rural Sylhet, Bangladesh: a cluster randomized trial. BMC Pregnancy Childbirth. 2015;15:326.
7. Ugwumadu A, Manyonda I, Reid F, Hay P. Effect of early oral clindamycin on late miscarriage and preterm delivery in asymptomatic women with abnormal vaginal flora and bacterial vaginosis: a randomised controlled trial. Lancet (London, England). 2003;361(9362):983-988.
